# Cognitive phylogenies, the Darwinian logic of descent, and the inadequacy of cladistic thinking

**DOI:** 10.3389/fcell.2015.00064

**Published:** 2015-10-14

**Authors:** Constantina Theofanopoulou, Cedric Boeckx

**Affiliations:** ^1^Department of General Linguistics, Universitat de BarcelonaBarcelona, Spain; ^2^Catalan Institute for Research and Advanced StudiesBarcelona, Spain

**Keywords:** cladogram, cognitive phylogeny, evolution, cognition, descent with modification

## Abstract

There has been a reappraisal of phylogenetic issues in cognitive science, as reconstructing cognitive phylogenies has been considered a key for unveiling the cognitive novelties that set the stage for what makes humans special. In our opinion, the studies made until now have approached cognitive phylogenies in a non-optimal way, and we wish to both highlight their problems, drawing on recent considerations in philosophy of biology. The inadequacy of current visions on cognitive phylogenies stems from the influence of the traditional “linear cladograms,” according to which every seemingly new or more sophisticated feature of a cognitive mechanism, viewed as a novelty, is represented as a node on top of the old and shared elements. We claim that this kind of cladograms does not succeed in depicting the complexity with which traits are distributed across species and, furthermore, that the labels of the nodes of these traditional representational systems fail to capture the “tinkering” nature of evolution. We argue that if we are to conceive of cognitive mechanisms in a multi-dimensional, bottom-up perspective, in accordance with the Darwinian logic of descent, we should rather focus on decomposing these mechanisms into lower-level, generic functions, which have the additional advantage of being implementable in neural matter, which ultimately produces cognition. Doing so renders current constructions of cognitive phylogenies otiose.

As de Waal and Ferrari (2010) observed, for the past few decades comparative cognitive research has focused “on the pinnacles of mental evolution,” asking “all-or-nothing questions such as which animals (if any) possess a theory of mind, culture, linguistic abilities, future planning, and so on.” De Waal and Ferrari remark that “research programs adopting this top-down perspective have often pitted one taxon against another, resulting in sharp dividing lines.” As a result, “insight into the underlying mechanisms has lagged behind (…).” Although de Waal and Ferrari add that “a dramatic change in focus now seems to be under way, …, with increased appreciation that the basic building blocks of cognition might be shared across a wide range of species,” we think that remnants of “top-down” thinking remain very influential to this day. We would like to show this by examining what we take to be the dominant conception of cognitive phylogenies, well-articulated in Fitch et al. (2010) (see also Hauser et al., 2002, and the numerous proposals concerning “proto-language” stages, usefully synthesized in Fitch, 2010). Specifically, we want to stress the tension between the information represented in these cognitive phylogenies and the bottom-up thinking that we believe captures the Darwinian logic of descent best.

We agree with de Waal and Ferrari that the “bottom-up perspective, which focuses on the constituent capacities underlying larger cognitive phenomena, is more in line with both neuroscience and evolutionary biology.” As such, the bottom-up perspective has greater integrative potential, and should be favored.

As Fitch et al. (2010) point out at the outset of their paper, domains like “language and social cognition” are “complex constructs, involving many independent cognitive mechanisms.” We could not agree more with them that in order to shed light on the evolution of such mechanisms, the comparative approach provides a powerful route. But in order to be successful, this approach must find the right level of comparative granularity, an issue that vitiates current attempts to construct cognitive phylogenies, for reasons we are about to discuss.

Ravignani et al. (2014) are right to stress that a monolithic viewpoint leads naturally to unhelpful questions, such as “when did language evolve?” (suggesting that this happened during one brief moment in human evolutionary history) or “where is language located in the brain?” (as if this complex cognitive ability occupies a single cortical region). Correctly, they see a “divide and conquer” strategy as the antidote to this monolithic conception: one must recognize that “any complex cognitive capability relies upon a suite of interacting cognitive capabilities. Each of these capabilities may have its own neural bases and independent evolutionary history.”

Although this “divide-and-conquer” strategy appears to be in line with de Waal and Ferrari's “bottom-up” approach, we don't think that they have been fully integrated with one another yet, because of how researchers continue to think of (cognitive) evolutionary novelties.

As can be gathered from the “proto-language” literature (first, the “lexicon” then “syntax,” as in the “lexical protolanguage” proposal; or first “sign” then “speech,” as in the “gestural protolanguage proposal' see Fitch, 2010 for extensive references), or even more clearly in Hauser et al. (2002) distinction between “the faculty of language in the broad sense” and “the faculty of language in the narrow sense,” current examples of cognitive phylogenies mostly tend to view every seemingly new or more sophisticated aspect of a cognitive faculty (properly decomposed) as a novelty, whose emergence leads to add a new ingredient (represented as a node) on top of the old and shared elements. Thus, Hauser et al. (2002)'s distinction between the Faculty of Language in the Broad and in the Narrow Sense was intended to make exactly this point, as non-shared elements were thought to bring out the element that led to the emergence of human language.

It is in this sense that current cognitive phylogenetic studies operate in opposition to both evolutionary thinking, which stresses descent, and also to contemporary attempts to ground cognitive capacities onto basic neurophysiological principles. These seek to provide a decomposition or fractionation of a particular cognitive domain into formal operations that are, in the words of Poeppel (2005), “elemental and generic.” Why should they be “elemental and generic”? Well, simply because this is the nature of the information coming from bottom-up approaches, grounded in what genes provide (Boeckx and Theofanopoulou, 2014, 2015). Since they are generic, these operations will be shared, across species, across cognitive domains, etc. As a result, they are not appropriate to draw cognitive phylogenies (i.e., to capture the cognitive branching off of species needed to adorn traditional phylogenetic trees with cognitive capacities). Conversely, the cognitive descriptions used to construct cognitive phylogenies are bound to retain a top-down, contrastive character.

To a certain extent, this last claim should not come as a surprise. After all, traditional representations of phylogenies, such as the only illustration gracing Darwin's Origin of Species, are inherently contrastive, as they focus on how species branched off from a common ancestor. Tree-like representations are meant to capture what made population A become distinct from population B, and when. Given that attempts to construct cognitive phylogenies boil down to attempts to graft cognitive traits onto independently established phylogenetic trees, as Fitch et al. (2010) made clear, it is to be expected that they too will tend to adopt a contrastive character, seeking to highlight a novel trait that makes the cognitive profile of population A different from that of population B.

But we very much doubt that cognition can be studied independently of the basic neurophysiological principles that produce it. As Tinbergen (1963) already stressed in his programmatic essay on ethology, no evolutionary adequate account of cognitive capacities can afford to ignore the brain mechanisms underlying mental capacities.

More and more, comparative approaches that adopt a bottom-up perspective reach the conclusion that “the distinction between general and (…) specialized mechanisms is hard to draw.” Indeed, “the distinction itself is of little use in furthering our understanding of the mechanisms” (Fitch, 2011). In other words, the more we learn about the mechanisms, and the more we set our focus on identifying them, the harder it becomes to capture cognitive specializations in a meaningful way, let alone represent them in a tree-like fashion. Put another way, the bottom-up perspective makes it hard to capture “cognitive speciation,” which is exactly what cognitive phylogenies represent.

To illustrate the tension that occupies us, consider briefly the following two examples. One shows how a shared brain mechanism can underlie distinct cognitive phenotypes; the other shows how the same phenotype can be subserved by distinct brain mechanisms. Both are equally problematic to draw cognitive cladograms, in as much as these are meant to remain true to the nature of cognition (which is brain-based).

The first example is the case of the speech rhythms found in humans. Following original insights from MacNeilage (collected in MacNeilage, 2008), as well as substantial progress in characterizing the brain basis of speech (Giraud and Poeppel, 2012), comparative research has focused on the phenomenon of lip smacking, an affiliative signal observed in many genera of primates. This facial expression exhibits a speech-like rhythm in the 3- to 8-Hz frequency range. Studies using developmental, x-ray cineradiographic, EMG, and perceptual approaches with non-human primates, reviewed in Ghazanfar and Poeppel (2014), converge on the hypothesis that the brain rhythm (“mechanism”) underlying lip-smacking was recruited for purposes of speech. Specifically, lip-smacking was linked to vocal output to produce the original rhythmic audiovisual speech-like utterances in the human lineage. Clearly, cognitively speaking, lip-smacking is not speech (Martins and Boeckx, 2014). Speech is in fact something that attempts to construct cognitive phylogenies would like to use to capture “cognitive speciation” events, as it were [think of Liberman's (1985) “speech is special” hypothesis, which continues to be at the heart of debates such as Hauser et al. (2002) vs. Pinker and Jackendoff (2005)]. But doing so would miss the neural basis of speech. Worse, the mechanism underlying lip-smacking itself (brain oscillation in the theta-frequency range) is not specific to this behavior (see Buzsáki et al., 2013 on the conservation of brain rhythms in mammals). As a result, we face a paradoxical situation when attempting to represent all this information in a tree-like format: speech is special, but the mechanism underlying it is deeply-rooted. Even if one appeals to another level of description to characterize speech (e.g., the “dynomic” description of theta-nested gamma oscillations), we still face the problem that these neural rhythms, and their nested relations, are shared across species and cognitive domains.

The second example comes from another domain of neuroethology. It concerns the representation of the environment from unreliable sensory cues, a brain function that is vital for survival. Humans and other animals use the interaural time difference (ITD) for sound localization. ITD is the difference in the arrival time of a sound at the ears. ITD results from unequal distances of a sound source to the two ears when the source is to the left or to the right of the listener. Grothe and colleagues have specified the mechanism of ITD in great neural detail in various species (see Grothe and Pecka, 2014 for an overview). We will not go into this here. Rather, what we want to stress is a finding reported in Lesica et al. (2010): For decades, it was assumed that the coding of ITDs in the mammalian brain was similar to that in the avian brain, where information is known to be sparsely distributed across individual neurons. Lesica et al. compare the representation of ITDs in adult male and female gerbils and in adult male and female barn owls. For gerbils, they used different decoders to infer ITDs from the activity of a population of neurons in central nucleus of the inferior colliculus. On the basis of this, they concluded that ITDs are not represented in a distributed manner, but rather in the summed activity of the entire population. The same analysis was performed on activity from the external nucleus of the inferior colliculus of adult male and female barn owls, which confirmed that in this case, ITDs were represented in a distributed manner. In sum, unlike the avian brain, the mammalian brain represents ITDs in the overall activity of a homogenous population of neurons within each hemisphere. Both the avian and the mammalian brains represent ITDs, so again we face a paradoxical situation. A cognitive phylogenetic tree would like to capture this similarity, but doing so would obscure the mechanistic differences underlying it.

Clearly, in both illustrations just discussed, it would be unsatisfactory to entertain distinct “cognitive phylogenies,” one capturing information at the phenotypic level, and another, information at the level of mechanism, for cognition characterizes itself by the integration of information from various levels. It cannot be dissociated from its underlying neural mechanisms. This is, we believe, the essential lesson of Marr (1982). Marr urged cognitive neuroscientists to combine (and not contrast) information from various levels (three levels for him; for a more refined view as to which levels should be taken on board, see Boeckx and Theofanopoulou, 2014; Figure 1).

**Figure 1:**
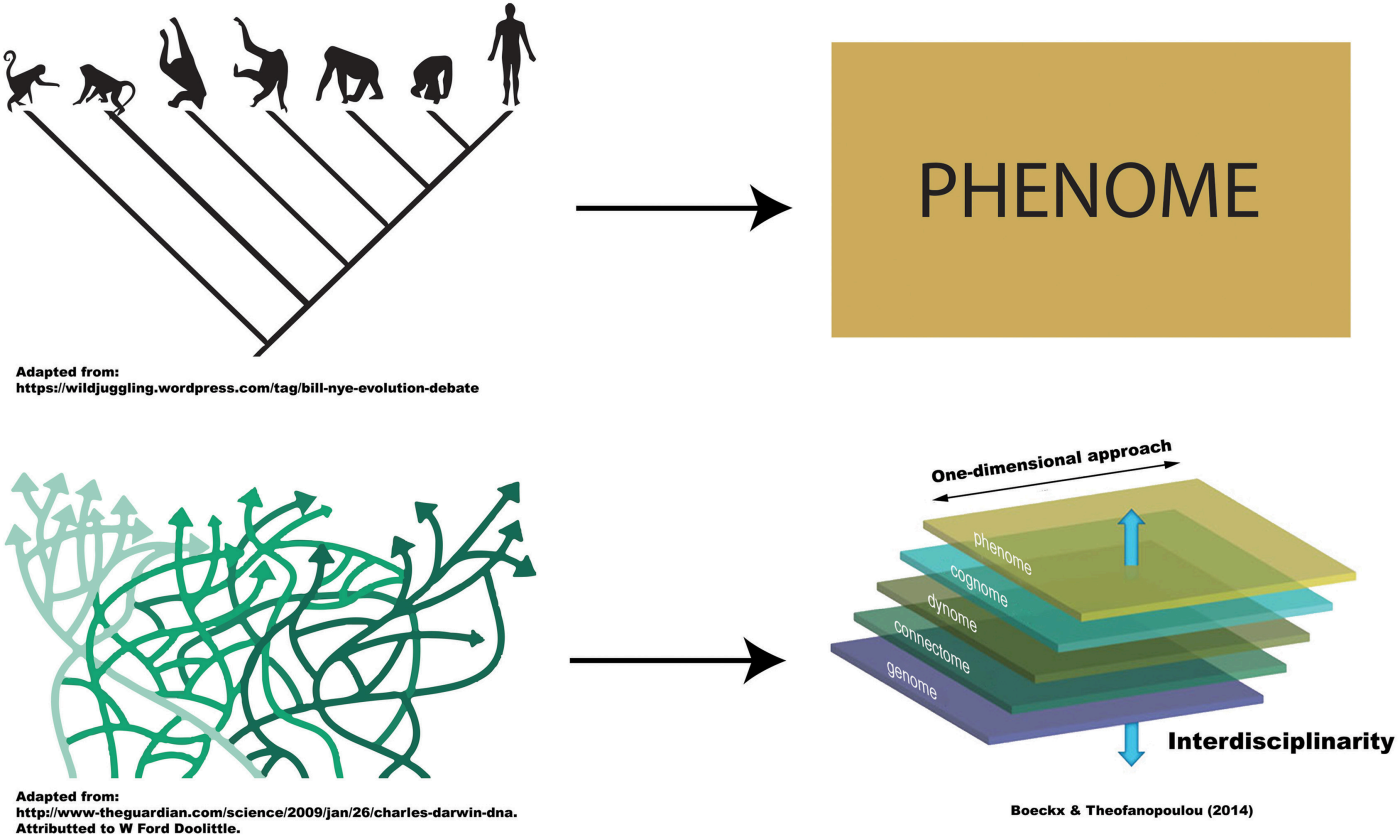
**Uni-dimensional vs. Multi-dimensional approaches to (cognitive) phylogenies**. The figure is meant to represent the fact that traditional, tree-like cognitive phylogenies can only be entertained if we adopt uni-dimensional approaches. As soon as we integrate information across levels of description (multi-dimensional approaches), the topology of cognitive phylogenies changes dramatically, to the point of being unhelpful to depict descent.

The larger point we want to make is that current attempts to construct cognitive phylogenies appear inherently incapable of capturing how evolution really works, specifically how novelties arise. Evolution does not work in a “yes or no” way, but rather tinkering what already exists, either transfiguring a system to give it a new function or modulating several systems to produce a more complex one. Of course, there is modification along the path of descent, but the modified aspect and the shared aspect are inextricably linked.

As discussed extensively in Balari and Lorenzo (2015) and the references cited therein (especially Wagner, 2014), the root of the problem lies in how one thinks of evolutionary novelties. Evolution, generally, and evolution of cognition, specifically, does not operate in a simplistic innovative way; rather, it reorganizes already existing generic mechanisms, recruiting them into new uses. We could liken the way that evolution works to “discovery” as opposed to “invention.” Novelty does not arise as a *de novo* invention, but as a tinkering-discovery of how generic operations could be combined, so that their collective effects stand as an apparent *de novo* trait [As a reviewer notes, this idea is very much in line with Reid's (2007) vision of evolution as “natural experiment”]. Classical cladograms, of the sort Fitch et al. (2010) borrowed to draw their cognitive phylogenies, fail to take into consideration evolution's essential tinkering nature. As a reviewer points out, it is worth bearing in mind that the aim of cladistics is, after all, to reconstruct natural relationships of organisms (not necessarily phylogenies) by means of certain key traits that are used for diagnostic purposes only. That is to say, cladistics is not originally intended as a model to trace the phylogeny of the corresponding traits (or lack thereof), let alone to speak to their mechanistic causes.

Another problem for current examples of cognitive phylogenies is that they appear to suffer from what Balari and Lorenzo (2013) called the “functionalist fallacy.” They focus on functions, but should also specify the mechanisms. Even if we decompose complex cognitive traits like language or social cognition, their component parts—altruistic behavior, empathy, mimicry vs. imitation, gaze following, and the like—still remain top-down terms. As de Waal and Ferrari (2010, p. 202) put it: “Outcomes are important from an evolutionary perspective in that they determine an organism's success at dealing with its environment, but from a cognitive perspective they are mere surface phenomena. Unique outcomes do not always reflect unique processes.” Indeed, inquiring whether a species performs or not a specific “outcome,” such as geometric gaze following or Theory of Mind (as suggested in Fitch et al., 2010) does not yield much insight into the processes by which every species independently comes to reach this very outcome. Therefore, we believe that asking yes/no questions makes us less likely to unveil the sub-processes that most probably lie beneath the emergence of cognitive profiles.

In this context it is worth bearing in mind Buzsáki's (2005) remark that it is a rather dangerous strategy for a research to start off from a man-created word or concept (he lists orientation, voluntary movement, and dead reckoning, in the context of spatial navigation, but virtually any node label of current cognitive phylogenies would do), because, in this way, it is likely to be limited to the “brain mechanisms that may be responsible for the generation of the conceived behavior.” Instead, the Darwinian logic of descent with modification demands a more widespread comparative basis. Also, the emphasis on mechanisms demands a move away from locationalist (neo-phrenologist) tendencies. As Poeppel (2012) put it, mapping is not explaining. Behaviors, especially complex ones, will have to be deconstructed, and reconstructed. One of the clearest cases in support of our view comes from Schaafsma et al.'s (2015) attempt to deconstruct and reconstruct the notion of Theory of Mind. The end result (captured in their Figure 3) is a complex web of properties that cannot possibly be mapped onto a standard cladogram.

There is an obvious sense in which current proposals concerning cognitive phylogenies continue to approach the mind in very modular terms, using fairly traditional categories (“syntax,” “phonology,” “semantics,” in the case of language) that are not the currency that the brain transacts in (Shalom and Poeppel, 2008). When trying to graft these traditional categories onto phylogenetic trees, cognitive scientists make the same neo-phrenologist mistake as neuroscientists trying to find “syntax” in the brain. We think this is seriously misguided.

Yet another problem for current models of cognitive phylogenies is that they do not abide by one very important characteristic of the cladograms of the Evolution of Life: they do not take the variable “time” as a common denominator. Phylogenetic trees are constructed according to two dimensions: species' phenotypes being one and time, the other. This does not work in the same way when taken to the level of cognition, for the reason that what these latter trees show is not the time a species took to reach a cognitive characteristic compared to another species. So grafting cognitive traits onto pre-established cladograms introduce an important temporal disconnect.

Obviously, we don't mean to discourage scientists from making phylogenetic hypotheses. Like many, we regard phylogenetic considerations as central to cognition. And we certainly don't want to suggest that the literature on proto-language or the evolution of cooperation, etc. have yielded no insights. The top-down perspective (and the cladistic representations it naturally gives rise to) has proven useful in identifying some key research areas and articulating important research questions. But we think it is time they be complemented with more bottom-up-oriented approaches to eventually get to the underlying mechanisms.

The pitfalls and limitations of traditional phylogenetic representations and tree-thinking are already well known to biologists. Thus, Omland et al. (2008) claim that traditional cladograms “misrepresent the evolutionary process.” Gregory (2008) warns that “any given node …represents a diverse assemblage …with a complex evolutionary history.” Degnan and Rosenberg (2009) insist that “conflicting genealogical histories” and “branching patterns” exist, but we feel that in the case of cognition, these are magnified. Species label may fit (more or less) into cladograms, but cognitive faculties don't. In line with recent approaches like neurodiversity that stress the “spectrum” character of variation (as do evo-devo approaches; cf. West-Eberhard, 2005), without any clear-cut divide, we think it important at this stage of research to stress descent at all possible levels of description. In other words, since we are at an early stage of research into these questions, there is still time to adopt more suitable perspectives, and to orient inquiry squarely toward brain-based hypotheses.

Likewise, evolutionary biologists have learned to conceive of “phenotypic novelty” as “largely reorganizational rather than a product of innovative genes” (West-Eberhard, 2005). Cognitive biologists ought to adopt this perspective as well (For relevant discussion, we again refer to Balari and Lorenzo, 2015).

In order to envisage cognitive phylogenies, we need to move away from attempts to graft cognitive notions onto cladograms, and instead decompose the cognitive mechanisms into (even) lower-level functions to trace back their ancestry and the ways in which these were mixed in novel ways. As de Waal and Ferrari (2010, p. 201) promote: “what if we were to replace our obsession with complex cognition with an exploration of basic processes? Instead of asking which species can do X, the question would become how does X actually work?” We suggest that we take as a starting point low-level circuit functions (e.g., gain modulation, phase coding, selective inhibition) that are instrumental for the mechanistic, neural implementation of cognitive functions, and we need to reason about these and how they combine to yield better-known cognitive traits (for a good example, see Bosman et al., 2014). Doing so may put us in a much better position to take advantage of the fact that the very fabric of neurophysiology (brain rhythms) is deeply conserved (Buzsáki et al., 2013). This very fact may well prove as central to cognitive biology as the deep conservation of hox-genes (deep homology) was for the evo-devo breakthrough.

## Funding

The present work was funded by a Marie Curie International Reintegration Grant from the European Union (PIRG-GA-2009-256413), research funds from the Fundació Bosch i Gimpera, the Generalitat de Catalunya (2014-SGR-200), and the Spanish ministry of economy and competitiveness (FFI2013-43823-P, FFI2014-61888-EXP).

### Conflict of interest statement

The authors declare that the research was conducted in the absence of any commercial or financial relationships that could be construed as a potential conflict of interest.
